# The Fabrication of Porous Metal-Bonded Diamond Coatings Based on Low-Pressure Cold Spraying and Ni-Al Diffusion-Reaction

**DOI:** 10.3390/ma15062234

**Published:** 2022-03-17

**Authors:** Zhicheng Zhang, Zhanqiang Liu, Hui Ge, Bing Wang, Yukui Cai, Qinghua Song

**Affiliations:** 1 School of Mechanical Engineering, Shandong University, Jinan 250061, China; sduzhangzhicheng@mail.sdu.edu.cn (Z.Z.); sduwangbing@sdu.edu.cn (B.W.); caiyukui@email.sdu.edu.cn (Y.C.); ssinghua@sdu.edu.cn (Q.S.); 2 Key Laboratory of High Efficiency and Clean Mechanical Manufacture of MOE/Key National Demonstration Center for Experimental Mechanical Engineering Education, Jinan 250061, China; 3 China National Heavy Duty Truck (Group Corp.), Jinan 250061, China; gehui@sinotruk.com

**Keywords:** cold spraying, post-spray heat-treatment, porous metal-bonded diamond coating, wear behavior

## Abstract

A porous metal-bonded diamond grinding wheel has an excellent performance in precision grinding. In this research, a novel manufacturing process of porous metal-bonded diamond coating was presented. Firstly, the diamond/Ni/Al coatings (400–600 μm) were fabricated via low-pressure cold spraying and their microstructures were studied. The diamond particles in the feedstock had a core–shell structure. Secondly, the post-spray heat-treatments were set at 400 °C and 500 °C to produce pores in the cold-sprayed coatings via Ni-Al diffusion. The porosities of 400 °C and 500 °C heated coating were 8.8 ± 0.8% and 16.1 ± 0.7%, respectively. Finally, the wear behavior of porous heated coating was tested in contrast with cold-sprayed coating under the same condition via a ball-on-disc tribometer. The wear mechanism was revealed. The porous heated coating had better wear performance including chip space and slight clogging. The surface roughness of wear counterpart ground by the porous heated coating was smaller (Sa: 0.30 ± 0.07 μm) than that ground by cold-sprayed coating (Sa: 0.37 ± 0.09 μm). After ultrasonic clean, the average exposure height of diamond particles in the wear track of porous heated coating was 44.5% higher than that of cold-sprayed coating. The presented manufacturing process can contribute to fabricate high performance grinding wheels via cold spraying and porous structure controlling through Ni-Al diffusion–reaction.

## 1. Introduction

Diamond has an ultra-high hardness, large bulk modulus, high thermal conductivity [1] and high abrasive ability [2]. It has been used to machine ceramics, composites, glass, non-ferrous metals, and so on [3]. The bond of diamond grinding wheel could be metal [4], ceramic [5] or resin [6]. The metal-bonded grinding wheel is widely used in accurate grinding and ultra-precision grinding due to good shape retention [7], good matrix strength, high holding force of abrasives, high wear resistance and good loading capacity [8].

The hot pressing sintering process has been used to fabricate metal-bonded diamond coatings [9]. Thermal spraying [10], laser cladding [11], laser-directed energy deposition [12], supersonic laser deposition [13] and other processes have also been studied. However, high working temperatures increase the risk of diamond graphitization [13], which will reduce the coating performance.

Tillmann et al. [14] manufactured metal-bonded grinding wheel via cold spraying. Therefore, cold spraying is a promising technology to fabricate a metal-bonded grinding wheel. As for cold spraying, micron-sized powders are accelerated to 300–1400 m/s [15] through De Laval nozzle by carrier gas. The powders impact the substrate surface and achieve deposition via severe plastic deformation [16]. The powders remain solid state without melting. Thereby, cold spraying avoids some problems such as oxidation, phase transformation and residual thermal stress [17]. In general, cold spraying can produce both thin (microns level) and thick coatings (centimeters level) [18,19]. Furthermore, the substrates for cold spraying can be metals, polymers [20], and ceramics [21]. According to the main gas pressure, cold spraying is divided into a high-pressure and low-pressure cold spraying system [22].

More research about cold-sprayed metal matrix composite (MMC) has emerged. As for metal-bonded diamond coating, to avoid the fracture of diamond particles in the cold spraying process and improve its content, some methods were put forward. For example, the original diamond particles are transformed into core–shell structure [23,24]. In addition, the core–shelled diamond was applied in the manufacturing of a grinding wheel [25]. For the grinding wheel, the grinding ability would reduce due to intensive clogging [26]. Various types of solutions were studied to reduce this unfavorable effect, such as the process of impregnation of abrasive tools with different types of substances [27]. However, the abrasive, bond, and porosity are dominant factors influencing the performance of grinding wheel [28]. The wheels with low porosity have the disadvantages such as small chip space, clogging, and bad self-sharpening ability [29], which cause the hard truing and dressing of the grinding wheel and the burn of work piece surface [30]. The porous metal-bonded grinding wheel has superiorities of self-sharpening capability [31]. The cold-sprayed metal-bonded diamond coatings have low porosity.

The cold-sprayed coating has a dense structure. Less pores may come from the incomplete bonding between particles [32] or any prior gas porosity trapped in the particles during the particle manufacturing process [33]. Moridi et al. [34] created porous cold-sprayed coatings by controlling surface temperature and processing conditions below the critical powder impact velocity. However, the feedstock was only metal material and the cold spraying conditions need be accurately controlled. Generally, post-spray heat-treatment of the coating can reduce the porosity due to the enhanced particle bonding at high temperature [35]. However, on the contrary, pores could be fabricated in the cold-sprayed coatings by post-spray heat-treatment according to Spencer and Zhang [36]. They aimed to fabricate metal matrix Ni-Al intermetallic reinforced composite, but in addition to Ni-Al intermetallic, pores were produced. Meanwhile, porous NiAl alloys could be prepared by die-pressing Ni + Al powder mixtures and vacuum sintering [37].

**Figure 1:**
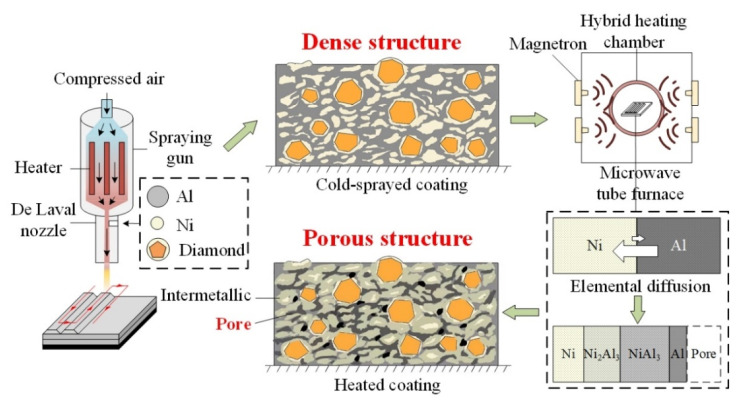
Fabricating porous metal-bonded diamond coatings based on low-pressure cold spraying and Ni-Al diffusion–reaction.

Therefore, this work aimed to fabricate porous metal-bonded diamond coatings, which can be used in precision grinding. Conventional cold spraying research rarely focused on the manufacturing of porous coatings. One of the criteria of the better coating quality is the low porosity. This work aimed to transform the cold-sprayed dense structure into porous structure. A new method to fabricate porous grinding wheel was proposed. The manufacturing process was put forward and shown in Figure 1. Firstly, the metal-bonded diamond coatings were fabricated by low-pressure cold spraying equipment. Secondly, the diffusion–reaction between Ni and Al was used to create pores in the coating through post-spray heat-treatment. Finally, the tribology tests of cold-sprayed coatings and porous heated coatings were carried out. The results showed the fabricated porous metal-bonded diamond coatings had better wear performance and large potential in precision grinding. The proposed manufacturing process would be promising to fabricate a porous grinding wheel. The porosity was controlled by adjusting Ni-Al diffusion–reaction. The content of Al, the post-spray heat-treatment temperature, and holding time need to be optimized to have better porosity and grinding performance.

## 2. Experiments

### 2.1. Material Preparation

**Figure 2:**
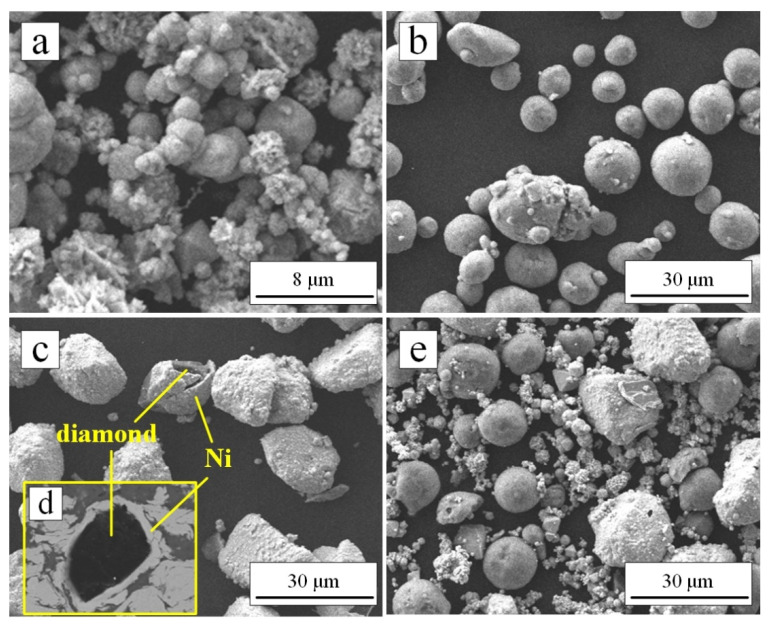
Morphology of the feedstock powders used in this study: (**a**) Ni, (**b**) Al, (**c**) diamond, (**d**) the core–shell structure of Ni-coated diamond and (**e**) the mixed powders.

The feedstock materials for cold spraying were listed in Table 1. The morphologies of the feedstock powders observed by scanning electron microscopy (SEM, JSM-7610F, Tokyo, Japan) were shown in Figure 2a–c. As shown in Figure 2c,d, the diamond particles had a core–shell structure, which consisted of two different layers: a diamond core and an electroless Ni outer-layer with a weight gain ratio of 100%. The Ni layer was believed to promote the bonding between diamond particle and coating. 

The content of the above powders was 42 wt.% (Ni), 16 wt.% (Al), and 42 wt.% (Ni-coated diamond). All powders were put into the nylon tank according to the above ratio, and zirconia balls were added (the mass ratio of the ball to the material was 1:6). A mechanical blending of powders was carried out using a powder mixer for 30 min at 1200 rpm. The mixed powders were shown in Figure 2e. It can be seen that the powders were mixed evenly. The YG 20 cemented carbide (WC, 78.90 wt.%; Co, 19.50 wt.%; Ni, 1.60 wt.%) was selected as the substrate. Before the spraying process, the substrate was grit-blasted with alumina powder (Grit 36) and ultra-sonically cleaned in absolute ethanol to enhance the adhesion between the coating and substrate.

### 2.2. Experimental Procedures

**Figure 3:**
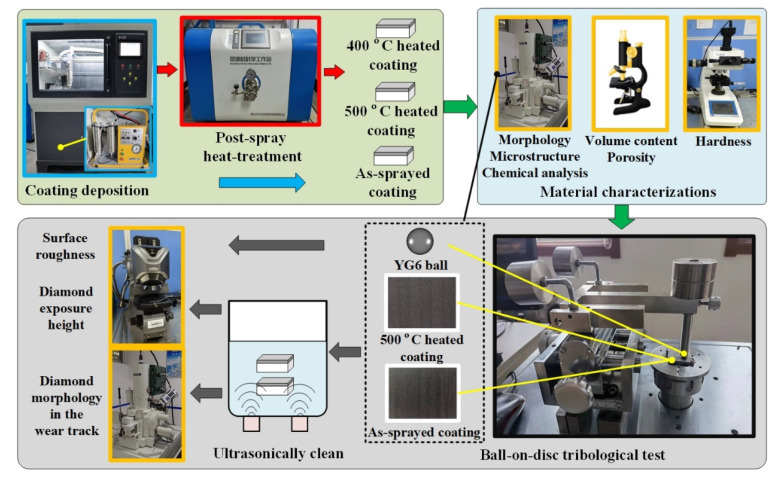
Experimental procedures and measurement schemes of fabricating and testing porous metal-bonded diamond coatings (heated coatings).

The experimental procedures and measurement schemes of fabricating and testing porous metal-bonded diamond coatings were shown in Figure 3. Firstly, the cold-sprayed coatings and porous heated coatings were prepared for observation and comparison. Then, the morphology, microstructure, composition content and phase transformation of coatings were analyzed. The porosities of porous heated coatings at different heat-treatment temperatures were measured. The hardness of cold-sprayed coatings and porous heated coatings was tested. Finally, the property of porous heated coating was tested via a tribology test compared with the cold-sprayed coating. The wear behaviors of porous heated coating and cold-sprayed coating were analyzed. The influence of porous structure on grinding performance was further explored. The detailed experimental procedures were described in the following text.

### 2.3. Coating Deposition

Low-pressure cold spraying equipment DYMET 423, produced in Obninsk Center for Powder Spraying, Obninsk, Russia, was used to deposit coating. It adopts the De Laval design with a converging-diverging nozzle. The nozzle throat diameter is 2.4 mm, the length of the divergent nozzle is 120 mm, and the exit diameter is 4.9 mm. The cold spraying deposition parameters were listed in Table 2.

### 2.4. Post-Spray Heat-Treatment

The as-sprayed diamond/Ni/Al coatings on YG 20 cemented carbide substrates were heat-treated at 400 °C and 500 °C, respectively, in high purity (≥99.999%) argon atmosphere (heating rate of 3 °C/min, holding time of 1 h, followed by furnace cooling) to form pores in the coating at a solid state. Post-spray heat-treatment was performed in a microwave tube furnace (WBMW-GW4, Tangshan Renshi huge source microwave apparatus Co., Ltd., Tangshan, China).

### 2.5. Material Characterizations

To assess the coating microstructure and perform chemical analysis, all samples were prepared using standard metallographic procedures, mounted first, followed by grinding and polishing with the final polishing applied by 0.25 μm diamond solution. The morphology of powder, the cross-section morphology and the microstructure of the coating were examined by scanning electron microscopy (SEM, JSM-7610F, Tokyo, Japan) in the secondary electron (SE) imaging mode and the back scattered electron (BSE) imaging mode. 

To examine the phase transformation of the composite coatings after cold spraying and post-spray heat-treatment, X-ray diffractometer (XRD, Smartlab, Tokyo, Japan) was used with Cu Kα at a current of 150 mA, a voltage of 40 kV and scan step of 0.02°. In addition, the energy dispersive X-ray analysis (SEM equipped with an EDS) was performed further to confirm the composition of intermetallic compounds in the coating. The volume contents of Ni, Al and diamond in the cold-sprayed coating and the porosity of heated coatings were calculated by ImageJ software (National Institutes of Health, v1.53c, Bethesda, America), using the cross-sectional images in the BSE imaging mode. Five images of cold-sprayed coatings and five images of heated coatings were used. The average values of volume content and porosity were gained respectively.

The microhardness of the coating was measured by a micro Vickers hardness tester (FM-800, Tokyo, Japan) with the load of 200 g for 15 s. During the selected tested points, the areas adjacent to the interface of coating/substrate, or the surface of coating and diamond were avoided. The indentations were taken at the Ni and Al matrix. The indentations were uniformly located 150–250 μm above the interface between the coating and the substrate to avoid hardness variation with gradient caused by work hardening [38]. Seven different positions of each coating were measured. The average value of microhardness was obtained by removing the maximum and minimum values in the test results.

### 2.6. Tribological Testing

The wear samples were cubic substrates and the coatings were deposited on flat surfaces. Therefore, the tribology tests were carried out with a ball-on-disc tribometer (MXW-005, Jinan, China) at room temperature (25 °C; humidity, 40–60%) to verify the property of fabricated porous metal-bonded diamond coatings. Before the test, the sample surfaces were polished to 1–2 μm roughness. The 6 mm diameter cemented carbide ball (YG6; WC, 94 wt.%, Co, 6 wt.%; 89.5 HRA) was used as the counterpart under a constant load of 4 N. The wear track radius was 5 mm, the rotation speed was 10 mm/s, and the duration was 30 min. 

The wear tracks of coatings were examined by SEM and EDS. The wear track morphology and the surface roughness of cemented carbide ball were constructed and measured by a 3D laser scanning microscope (VK-X200 series, KEYENCE, Osaka, Japan). Five different positions were measured to get the surface roughness and to derive the average value. After the tribology test, the blockage of coatings was checked after ultrasonically cleaning in absolute ethanol for 10 min. The diamond particles in the wear track were photographed by SEM. The exposure height of diamond particles in the wear track was measured by a 3D laser scanning microscope. The average and maximum exposure height of diamond particles were measured at five different positions. The average value of measured results was taken as the final result.

## 3. Results and Discussion

### 3.1. Microstructure Characterizations of As-Sprayed Coatings

**Figure 4:**
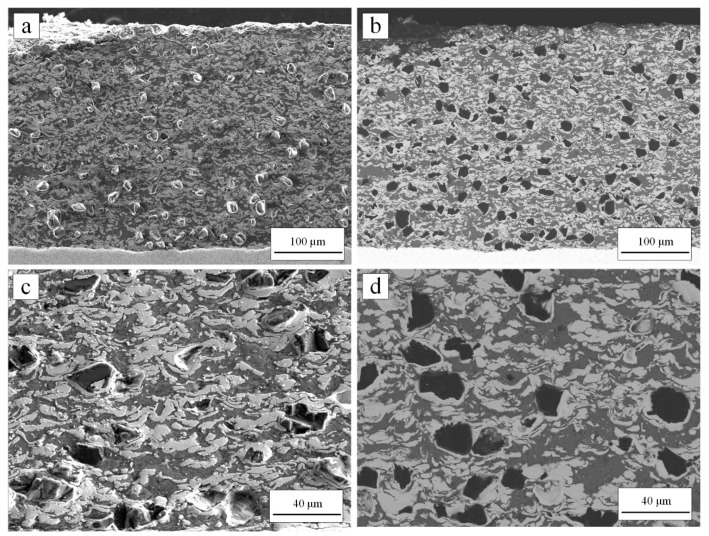
SEM images of the cross-section of diamond/Ni/Al coating at the magnification of 150× (**a**,**b**), and 500× (**c**,**d**): (**a**,**c**) were taken in SE imaging mode, showing the morphology of the cross-section; (**b**,**d**) were taken in BSE imaging mode, showing the content and distribution of diamond/Ni/Al.

**Figure 5:**
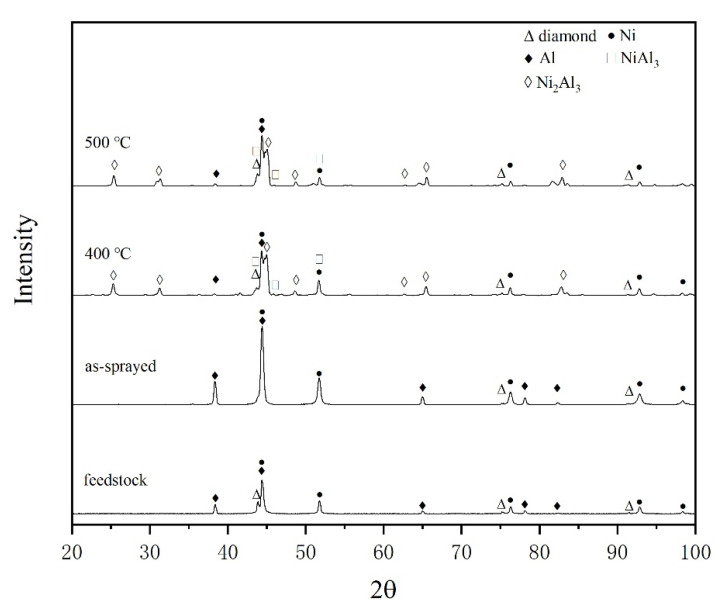
XRD patterns of feedstock, as-sprayed coating, heat-treated coatings at 400 °C and 500 °C.

The cross-sections of as-sprayed diamond/Ni/Al coatings were shown in Figure 4. The coatings were dense, and the thickness was 400–600 μm, with no delamination or cracks found in coatings. The diamond particles in Figure 4c–d were approximately the same size as the original diamond particles, which meant that the fracture of diamond particles during deposition was reduced compared with conventional cold spraying [39]. This was because the diamond particles were coated with a metal layer, which played a buffer role in the deposition process [24,40]. Meanwhile, the gas pressure of the low-pressure cold spraying system was lower than a high-pressure cold spraying system, resulting in a relatively lower particle velocity. The lower particle velocity can alleviate the fracture of diamond particles. In addition, XRD results in Figure 5 showed no graphite peak of as-sprayed coatings. Therefore, no graphitization happened during deposition.

**Figure 6:**
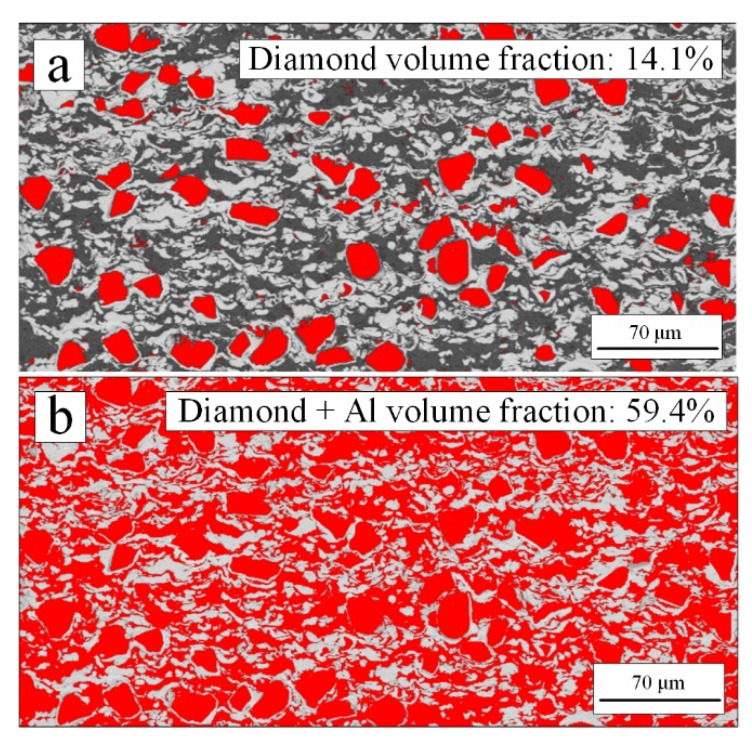
Content of diamond, Ni and Al in the coating analyzed by ImageJ software: (**a**) the diamond was selected by adjusting the threshold of binary image, then the content of diamond was calculated; (**b**) the diamond and Al was selected by adjusting the threshold of binary image, then the content of them was calculated.

Diamond, Ni, and Al were evenly distributed without single material aggregation according to Figure 4a–b. In addition, XRD results in Figure 5 showed that no Ni-Al intermetallic compound was formed during coating deposition. Figure 4 shows that the volume content of Ni, Al and diamond was obtained by processing the cross-sectional coating image in BSE mode through ImageJ software based on binary image analysis [23,41]. By adjusting the threshold, as shown in the red area in Figure 6, diamond area (Figure 6a), diamond and Al area (Figure 6b) were selected successively, then the volume content of each component in the coating was obtained.

The results compared with the feedstock were listed in Table 3. According to the results in Table 3, the diamond content decreased, still reaching the volume content of nearly 15%. For cold spraying, diamond particles with core–shell structure can increase the content of diamond in the coating [41,42]. The volume content of Ni and Al increased. The volume content ratio of Ni/Al had still changed from 1.20 (feedstock) to 0.88 (coating), which proved that the deposition efficiency of Al with a larger diameter was higher than that of Ni with a smaller diameter.

### 3.2. Microstructure Characterizations of Heat-Treated Coatings

Spencer and Zhang [36] reported when the cold-sprayed Ni + Al coating was heat-treated at 420 °C, and diffusion–reaction will occur between Ni and Al, resulting in the formation of Ni-Al intermetallic compound and porosity. Lee et al. [43] also reported that many pores were observed in the Al-Ni composite coating when annealed at 600 °C. The higher the annealing temperature, the more pores in the coating. Therefore, it would be possible to produce pores in the as-sprayed coating by post-spray heat-treatment at 400 °C and 500 °C, respectively.

**Figure 7:**
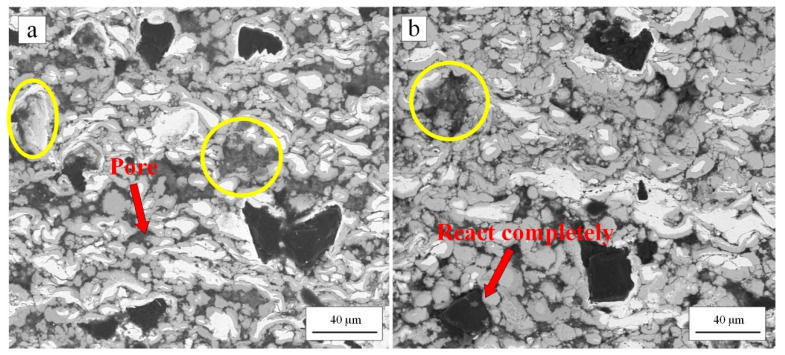
Backscattered SEM images of the cross-section of diamond/Ni/Al coating after 400 °C (**a**) and 500 °C (**b**) heat-treatment.

**Figure 8:**
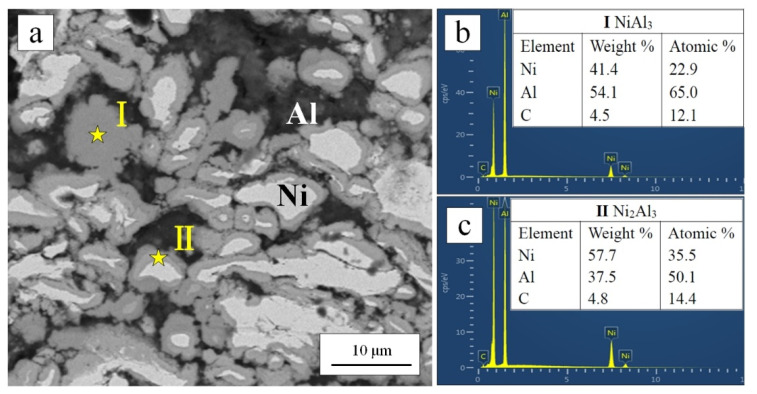
Backscattered SEM images of the cross-section of diamond/Ni/Al coating after 400 °C heat-treatment and the selected point (Ⅰ) and point (Ⅱ) at Ni-Al reaction products for EDS test (**a**); (**b**,**c**) showing the EDS results of the selected points.

The cross-section of the coatings heated at 400 °C was shown in Figure 7a. The morphology has changed greatly compared with the as-sprayed coating. It can be seen that the metal matrix was no longer dense, and there were many pores in the metal matrix. With Ni particles as the center, one or two layers of intermetallic compounds with different colors were formed around each Ni particle, and some Ni particles were completely reacted. According to the EDS analysis results in Figure 8, the reaction products were composed of Ni and Al elements. A small amount of C element was also detected at point (Ⅰ) and point (Ⅱ) with almost the same content. The C element came from the diamond solution used in the standard metallographic procedures. For point (Ⅰ) (dark gray region), the atomic ratio of Ni and Al was close to 1:3. For point (Ⅱ) (light gray region), the atomic ratio of Ni and Al was close to 2:3. Combined with XRD results in Figure 5, it can be further determined that the reaction products were NiAl_3_ (point (Ⅰ)) and Ni_2_Al_3_ (point (Ⅱ)). The composition of intermetallic compounds was consistent with the result reported by Spencer and Zhang [36].

The cross-section of the coatings heated at 500 °C was shown in Figure 7b. The diffusion–reaction degree between Ni, Al matrix, and the Ni layer of the diamond was higher. The pores were more obvious. EDS and XRD results showed that the reaction products were still NiAl_3_ and Ni_2_Al_3_. The XRD patterns of 400 °C and 500 °C heat-treated coating were similar. However, the intensity of elemental Ni and Al peaks of 500 °C heat-treated coating was lower than 400 °C heat-treated coating. This was consistent with a higher degree of diffusion–reaction of 500 °C heat-treated coating.

Pores were successfully produced through the Ni-Al in-situ reaction at a solid-state. With the increase of heat-treatment temperature, there were more pores in the coating. In addition, the uniform distribution of Ni and Al particles favored the pores evenly distributed in the coating. According to the test results, when the heat-treatment temperature was higher than 400 °C and the heating rate was 3 °C/min, Ni and Al can undergo solid-state diffusion–reaction at temperatures lower than the eutectic temperature (Ni/NiAl_3_, 640 °C). The causes of pore formation were classified into two points. Firstly, the higher density of intermetallic compounds than the average bulk density of as-sprayed coating [36]. Secondly, the diffusivity between Ni and Al was unbalanced when diffusion occurred at a solid-state [44]. The element Al has a higher diffusivity. A flux of vacancies went into the element Al due to the Kirkendall effect, which later condensed to form pores [36]. Therefore, pores formed on the original Al position. According to the test results, the porous structure could be controlled by adjusting the Al content [37] and post-spray heat-treatment.

However, some diamond particles fell off from the heated coatings after standard metallographic procedures as shown in the marked position (yellow coil) in Figure 7. According to the place marked by red arrow in Figure 7b, the diffusion–reaction may include the Ni outer-layer of core–shelled diamond particle. When the Ni outer-layer reacted completely, the diamond particle was separated from its core–shell structure due to the disappearance of an Ni outer-layer. In addition, pores were formed around diamond particles, which further reduced the bonding between the surrounding metal matrix and diamond particles. Therefore, such diamond particles with high exposure height fell off after standard metallographic procedures.

The microhardness of the coating cross-sections and the porosity of coatings at different conditions are listed in Table 4. Due to the staggered distribution of Ni and Al and the small diameter of Ni particles, the indenter was pressed on both the Ni and Al matrix every time during the hardness test. Therefore, the test results were higher than Al but lower than Ni. For the as-sprayed coatings, no obvious pores existed in Figure 4. Therefore, compared with the heated coatings, the porosity of as-sprayed coatings was negligible and can be defined as “tiny”.

After heat-treatment at 400 °C, although heat-treatment will cause the softening phenomenon of recovery and recrystallization [45], the hardness of the coating was further improved due to the formation of a large number of Ni-Al intermetallic compounds with higher hardness. However, it decreased at 500 °C heat-treated coating. For the 500 °C heat-treated coating, more pores reduced the ability of the coating to resist indenter pressing than 400 °C heat-treated coating.

### 3.3. Wear Behavior of As-Sprayed and Porous Heat-Treated Coatings

The 500 °C heat-treated coating had more pores than the 400 °C heat-treated coating. Therefore, 500 °C heat-treated coating and as-sprayed coating were selected to find the effectiveness of pores. 

**Figure 9:**
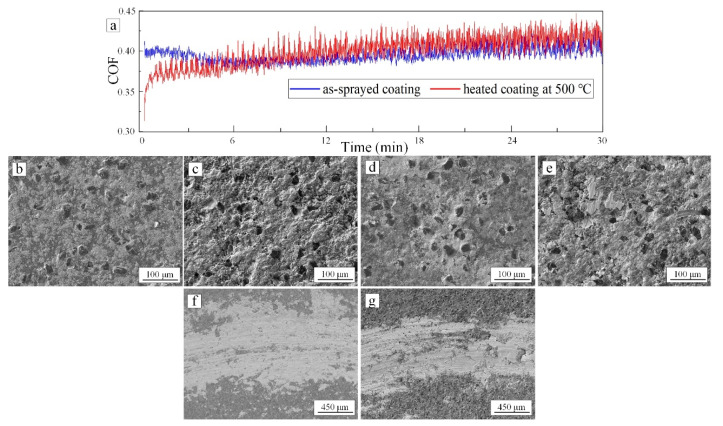
COF versus wear time for as-sprayed coating and 500 °C heat-treated coating (**a**); SEM images of the surfaces of the coatings at different stages (**b**–**g**); (**b**,**c**) original surfaces before the tribology test; (**d**,**e**) surfaces during the tribology test; (**f**,**g**) final surfaces after the tribology test; (**b**,**d**,**f**) corresponding to the as-sprayed coating and (**c**,**e**,**g**) corresponding to 500 °C heat-treated coating.

The coefficient of friction (COF) curves of the as-sprayed coating and the 500 °C heat-treated coating were shown in Figure 9a. Except for the differences in the initial stage, they were almost the same in the following stage. Both tended to be stable. In the initial stage, compared with the 500 °C heat-treated coating, the COF of the as-sprayed coating first decreased slightly. For the 500 °C heat-treated coating, the initial COF was lower than the former.

The surfaces of the as-sprayed coating and the 500 °C heat-treated coating corresponding to the different stage of the tribology test were shown in Figure 9b–g. Before the tribology test, due to the falling off of the diamond particles on the surface of 500 °C heat-treated coating, the amount of diamond particles was less than as-sprayed coating by comparing Figure 9b,c. Many pores existed on the surface of 500 °C heat-treated coating in Figure 9c. According to Figure 9d, light-colored debris were accumulated around diamond particles in the wear track of as-sprayed coating during the tribology test.

**Figure 10:**
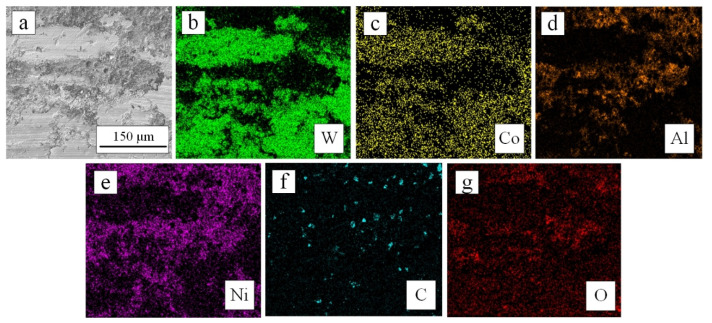
(**a**–**g**) SEM image and EDS mapping on the worn surface of a 500 °C heat-treated coating after tribology test.

After the tribology test, the wear tracks on both surfaces were covered by a light colored thin film in Figure 9f,g. It was continuous on the as-sprayed coating, but on the 500 °C heat-treated coating, part of the wear track was not covered by the film, and the film was relatively thin. According to the EDS analysis results in Figure 10, the light-colored thin film was a composite of W and Co, which were constituent elements of the cemented carbide ball. Therefore, due to the hardness of the diamond being higher than that of cemented carbide, resulting in the abrasion of the cemented carbide ball surface, the debris of a cemented carbide ball fell off during the tribology test and adhered to the coating surface [23,24]. In addition, according to the EDS analysis results, the O element was detected on the Ni and Al matrix; that is, oxide film was formed on the Ni and Al matrix surface.

Therefore, the wear mechanism of as-sprayed and 500 °C heat-treated coating can be summarized as abrasive and adhesive wear. At the beginning, for the as-sprayed coating, there were more diamond particles involved in grinding a cemented carbide ball than 500 °C heat-treated coating as shown in Figure 9b, resulting in a relatively high COF. Then, the exposed diamond particles were gradually adhesive by the cemented carbide debris, as shown in Figure 9d, which affected the grinding of cemented carbide balls and reduced the COF slightly. For the 500 °C heat-treated coating, there were pores on the coating surface. The cemented carbide debris generated from the tribology test filled the pores firstly. In the following stage, the debris gradually covered both as-sprayed and 500 °C heat-treated coating surface and formed a film. The COF of as-sprayed and 500 °C heat-treated coating tended to be stable in the friction process between the film and the worn surface of a cemented carbide ball.

**Figure 11:**
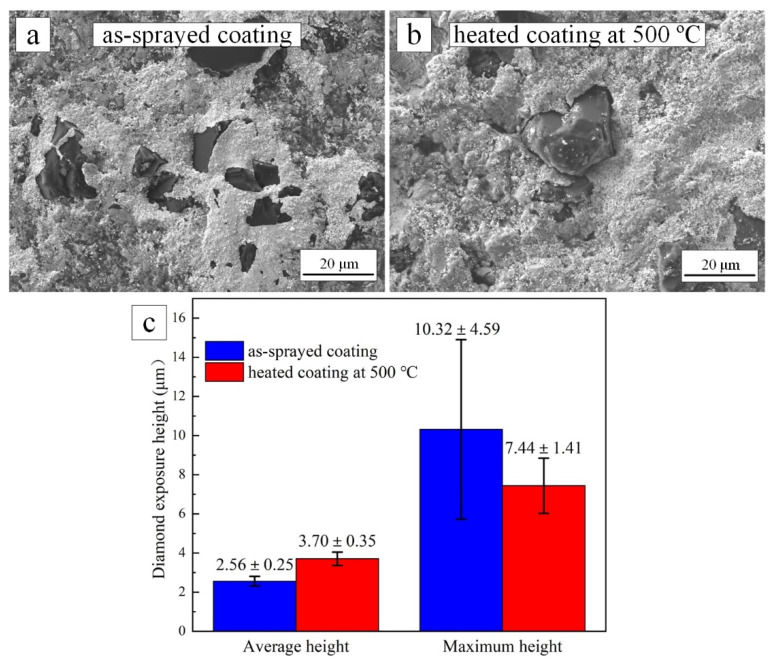
SEM images of the diamond particles in the wear track of as-sprayed coating (**a**) and 500 °C heat-treated coating (**b**) after ultrasonic cleaning; the exposure height of diamond particles measured by a 3D laser scanning microscope (**c**).

The test results proved that both as-sprayed and 500 °C heat-treated coating could grind the cemented carbide grinding ball. However, porous structure played an important role in the accommodation of debris. The SEM images of the diamond particles in the wear track after ultrasonic clean were shown in Figure 11. The diamond particles of the as-sprayed coating were adhesive by cemented carbide debris in Figure 11a. This corresponded to the blockage of the grinding wheel [46]. The diamond particles of the 500 °C heat-treated coating were slightly adhesive by cemented carbide debris in Figure 11b. The exposure height of diamond particles in the wear track was shown in Figure 11c. The average exposure height of diamond particles in the 500 °C heat-treated coating was about 44.5% higher than that in the as-sprayed coating. However, there were diamond particles with high exposure height in the wear track of the as-sprayed coating. 

**Figure 12:**
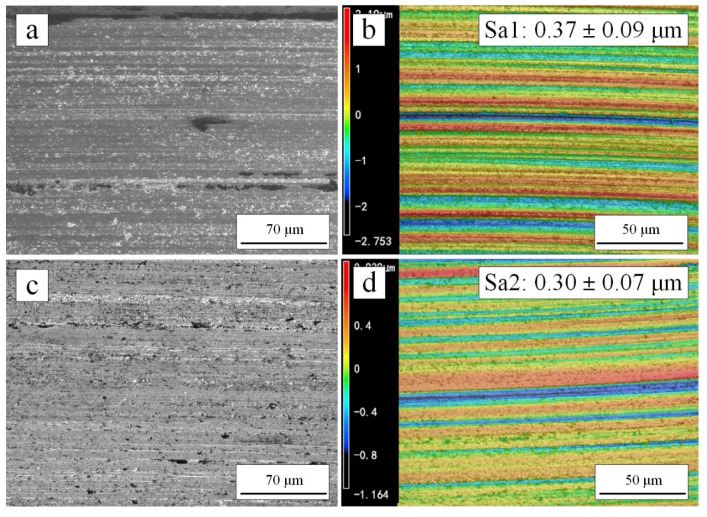
SEM images (**a**,**c**) and 3D profiles (**b**,**d**) on the worn surfaces of a cemented carbide ball after a tribology test: (**a**,**b**) corresponding to as-sprayed coating; (**c**,**d**) corresponding to 500 °C heat-treated coating.

More debris space and less blockage could favor the grinding performance. The worn surfaces of cemented carbide balls were shown in Figure 12. Both surfaces exhibited some distinct scratches due to specific protruding grains in the surfaces of coatings [14]. However, the cemented carbide surface ground by 500 °C heat-treated coating was smoother. According to the 3D contour scanning results in Figure 12b,d, the cemented carbide surface ground by 500 °C heat-treated coating had smaller surface roughness (Sa: 0.30 ± 0.07 μm) than the worn surface ground by as-sprayed coating (Sa: 0.37 ± 0.09 μm). Although the surface roughness of both was very small, the grinding performance of 500 °C heat-treated coating was better.

Therefore, 500 °C heat-treated coating had better wear performance. Due to the porous structure, the coating had chip space and slight clogging. It was easy for the diamond particles to emerge [7]. As a result, the cemented carbide surface ground by 500 °C heat-treated coating had smaller surface roughness.

## 4. Conclusions

In this paper, the porous metal-bonded diamond coatings were fabricated via low-pressure cold spraying and Ni-Al diffusion–reaction. This work aimed to transform the cold-sprayed dense structure into porous structure. This novel manufacturing process can contribute to fabricating high performance grinding wheels via cold spraying and porous structure controlling through an Ni-Al diffusion–reaction. The optimization of porous metal-bonded diamond coatings should be performed. Due to the different content of Al between feedstock and coating, the temperature and holding time of post-spray heat-treatment should be studied to have better control in porous structure. The main conclusions of this paper were as follows:

1. Diamond/Ni/Al coating was successfully deposited on the YG 20 substrate. The coating was thick (400–600 μm) and dense. Cold spraying could avoid the graphitization of diamond and the Ni-Al diffusion–reaction in the deposition process;
2. Pores were successfully produced at the Al site through the Ni-Al in-situ reaction at 400 °C and 500 °C, respectively. The porosities of 400 °C and 500 °C heat-treated coating were 8.8 ± 0.8% and 16.1 ± 0.7%, respectively;
3. Both cold-sprayed coating and 500 °C heat-treated coating showed the performance of grinding cemented carbide during the tribology test. The wear mechanism changed from coating worn by cemented carbide to the cemented carbide ground by the coating;
4. The porous structure of 500 °C heat-treated coating could benefit the wear performance in the tribology test. The porous coating had large chip space and slight clogging. The surface roughness of cemented carbide ground by 500 °C heat-treated coating was smaller (Sa: 0.30 ± 0.07 μm) than that ground by cold-sprayed coating (Sa: 0.37 ± 0.09 μm). After ultrasonic cleaning, the average exposure height of diamond particles in a wear track of the 500 °C heat-treated coating was 44.5% higher than that of the cold-sprayed coating.

## Figures and Tables

**Table 1:** Composition of feedstock for cold spraying.

Type	Size (μm)	Shape	Supplier
Ni (99.99%)	−10 + 5	Irregular morphology	Nangong Xindun Alloy Welding Material Spraying Co. Ltd., Xingtai, China
Al (99.98%)	d10 = 13	Spherical morphology	Henan Yuanyang Powder Technology Co. Ltd., Xinxiang, China
	d50 = 20		
	d90 = 30		
Ni-coateddiamond	diamond core	Irregular morphology	Henan Ruizhong New Material Technology Co., Ltd., Zhengzhou, China
	d10 = 20		
	d50 = 28		
	d90 = 38		

**Table 2:** Cold spraying deposition parameters.

Parameter	Value
Process gas	Compressed air
Powder feeding gas	Compressed air
Process gas pressure	0.7 MPa
Temperature	600 °C
Standoff distance	10 mm
Traverse speed	10 mm/s
Number of gun passes	4

**Table 3:** Ni, Al, and diamond content in the coating analyzed by ImageJ software.

Composition	Volume Content in the Feedstock	Volume Content in the Coating
Ni	37.3%	39.9 ± 1.9%
Al	31.2%	45.4 ± 2.3%
diamond	31.5%	14.7 ± 1.1%

**Table 4:** Microhardness of the metal matrix in the coating and porosity in different conditions.

	As-Sprayed HV_200_	Heated at 400 °C HV_200_	Heated at 500 °C HV_200_
Hardness	144 ± 17	179 ± 13	168 ± 10
Porosity	tiny	8.8 ± 0.8%	16.1 ± 0.7%

## Data Availability

Not applicable.
